# Prognostic and Immunological Value of GNB4 in Gastric Cancer by Analyzing TCGA Database

**DOI:** 10.1155/2022/7803642

**Published:** 2022-06-16

**Authors:** Binghui Liu, Lingbin Chen, He Huang, Huifeng Huang, Hui Jin, Chenglin Fu

**Affiliations:** ^1^Department of Pathology, Taizhou First People's Hospital, Huangyan Hospital of Wenzhou Medical University, Taizhou, Zhejiang, China; ^2^Department of Gastroenterology, Taizhou First People's Hospital, Huangyan Hospital of Wenzhou Medical University, Taizhou, Zhejiang, China

## Abstract

**Background:**

Gastric cancer (GC) represents a universal malignant tumor of the digestive system. Stromal and immune cells belong to two main nontumor components exerting a vital function in the tumor microenvironment.

**Methods:**

Based on TCGA database, this study downloaded clinical information and gene profiles of GC. The ESTIMATE algorithm was adopted for evaluating the score of immune-infiltrating cells. This work employed Sangerbox to explore the differentially denoted genes (DEGs) related to stromal, immunity, and prognosis. Besides, the STRING database was involved in order to detect the association among the proteins. The MCODE module of Cytoscape software was used to screen key genes. Oncomine and GEPIA databases were used, aiming to study the differences in key genes in healthy gastric mucosa and GC. At last, we adopted TISDIB and TIMER databases for analyzing the association of guanine nucleotide binding protein subunit-4 (GNB4) between gastric cancer and tumor immune cells. qRT-PCR was applied for exploring differential GNB4 expression between GC and normal gastric mucosa and investigating the relation of GNB4 with tumor-infiltrating lymphocytes (TILs).

**Results:**

Patients undergoing a great stromal score exhibited worse prognostic outcome, and cases having a low immune score had better prognosis. Overall, altogether 656 genes were upregulated with 5 genes being downregulated, which were matrix immune-related differential genes. Furthermore, 18 genes were screened as hub genes on the basis of the univariate Cox risk model of TCGA database (82 differential genes predicted poor GC survival). Oncomine and GEPIA databases revealed that GNB4 expression in gastric cancer was obviously higher in comparison with that in normal gastric mucosa. The GSEA, TISDIB, and TIMER databases revealed that GNB4 is involved in various tumor signal pathways and immune and metabolic processes. qRT-PCR demonstrated that GNB4 expression in gastric cancer was notably higher in comparison with that in normal gastric mucosa, showing significant association with matrix TILs.

**Conclusion:**

The selected key gene GNB4 is a potential biomarker to guide the immunotherapy of gastric cancer.

## 1. Introduction

Gastric cancer (GC) ranks the 5^th^ place among cancers and takes the 3^rd^ place among familiar reason for cancer-related mortality worldwide [1]. In addition, both the incidence and mortality rates are decreasing because of the research on risk factors and improvements in the techniques for early detection, surgical techniques, radiotherapy, and chemotherapy. However, its overall survival rate remains extremely low (31% in the United States and 25% worldwide) [2]. The main reason for the low survival rate is that most patients remain in the advanced stage at definitive diagnosis. Its main characteristics are high metastasis, high tumor heterogeneity, and chemoresistance [3]. According to the alterations of age groups together with the increasing world population, newly diagnosed GC patients and GC-related death cases globally will reach 7.5 million and 5.6 million, increasing by 58% and 73%, separately, by 2040 [4]. Hence, there is a great requirement to detect novel underlying biomarkers for the targeted treatment and identification of gastric cancer.

As a complex network, tumor microenvironment (TME) comprises a variety of tumor-related cells, like cancer stem cells (CSCs), cancer-associated fibroblasts (CAFs), MSCs, tumor-related inflammatory and immune cells, cancer-related adipocytes, pluripotent matrix cells, and erythrocyte cycle cells as well as endothelial cells (EC_S_) [5]. The above-mentioned cells can be primarily categorized into the following two groups, respectively, immune cells and stromal cells [6]. Several researches have pointed out the significance of these cells in the biology and microbial environment of different types of cancer [7–9]. The ESTIMATE algorithm can predict tumor purity together with infiltrating matrix/immune cell proportions in tumor tissues. To present stromal/immune cell rates, this study attempts to apply the immune and stromal scores [10]. It has been widely used in diverse tumors like breast, bladder, pancreatic, and lung cancers [11–14], and its effectiveness has been confirmed in studies.

**Figure 1:**
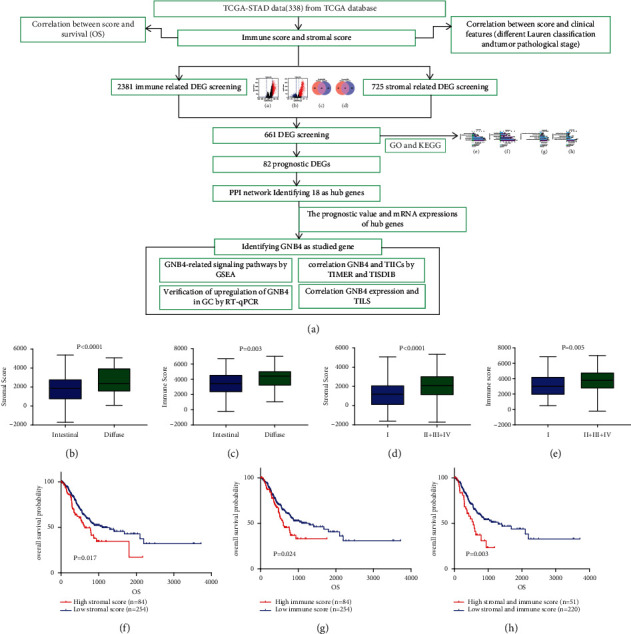
Stromal and immune scores are associated with the clinical features of gastric cancer and their overall survival. (a) Flow diagram of data preparation, processing, analysis, and validation in this study. (b) Distribution of stromal scores of gastric cancer between different Lauren classifications. (c) The correlation between stromal scores and tumor pathological stage. (d) Distribution of immune scores of gastric cancer between different histologic diagnosis. (e) The correlation between immune scores and tumor pathological stage. (f) Kaplan–Meier survival curve for patients with low vs. high stromal scores. (g) Kaplan–Meier survival curve for patients with low vs. high immune scores. (h) Kaplan–Meier survival curve for patients with low vs. high both stromal and immune scores.

We obtained the immune and stromal scores of gastric cancer in the present work based on ESTIMATE algorithm. In order to detect the genes with a prognostic value using the stromal immune score in gastric cancer, we could obtain gene expression profiles in The Cancer Genome Atlas (TCGA), deeply confirming association of gene level with patient prognosis in the Kaplan–Meier Plotter database. We also used GEPIA and Oncomine databases for assessing hub gene levels within healthy and tumor samples. One central gene, namely, guanine nucleotide binding protein subunit-4 (GNB4), was verified as a prognosis-related biomarker of gastric cancer and further analysed. TISDIB and TIMER databases were used to comprehensively study the state of GNB4 in gastric cancer-infiltrating immune cells in gastric cancer microenvironment. To conclude, the results indicated that GNB4 may become a different prognostic biomarker and therapeutic target in gastric cancer immunotherapy. We present the following article following the REMARK reporting checklist.  Figure 1(a) shows the workflow of this study.

## 2. Materials and Methods

### 2.1. Database and Assessment of Stromal and Immune Scores

We downloaded gene expression profiles of gastric cancer and clinical dataset from TCGA database (https://cancergenome.nih.gov/), which included age, gender, TNM stage, pathological classification, and survival time. Patients with survival time of ≤30 days (which may be because of other factors) and those without survival information were excluded from further evaluation. Finally, 338 patients were involved in the current work (Table 1). We shifted RNA-seq data for each case into transcription (TPM) values for normalisation. The ESTIMATE algorithm was adopted for the downloaded gene levels, for acquiring immune/stromal scores of each sample. The quartile method was applied to divide the stromal immune score of TCGA gastric cancer samples into high (quartile) or low (quartile 1–3) stromal/immune score group.

TCGA provided clinical data and gene levels of a total of 338 patients with gastric cancer. Among these patients, 218 (64.5%) were men and 120 (35.5%) were women. The mean age of those patients suffering from initial pathological diagnosis was 65.3 years (within the range from 35 to 86 years). Histopathological diagnosis contained 271 (80.2%) cases of intestinal adenocarcinoma and 67 (19.8%) cases of diffuse adenocarcinoma. Regarding the clinical stage of the tumor, 46 (13.6%) cases were presented in stage I, 278 (82.3%) cases were presented in stages II + III + IV, and 14 (4.1%) cases were in unknown stage. Based on ESTIMATE method, we obtained the stromal score (range: −1731.13 to −5372.17) and immune score (range: −224.39 to −7005.33) of all these GC cases.

### 2.2. Identification of Differentially Expressed Genes (DEGs)

After grouping high and low scores and TPM expression profile dataset for TCGA, we used limma [15] to screen differentially expressed genes (DEGs) in order to acquire those with |log2 (fold change)| > 1.5 (stromal group) or >0.5 (immune group). *P* < 0.01 served as a cut-off point to screen DEGs. A DEG volcano map was generated using Sangerbox software (http://vip.sangerbox,com), and we also plotted Venn diagram at Venny2.1.0 website (https://bioinfogp.cnb.csic.es/tools/venny/index.html).

### 2.3. Functional Enrichment Analysis of DEGs

We applied FunRich 3.1.3 [16] to perform functional annotation of DEGs, which was aimed at detecting GO categories by biological process (BP) and molecular function (MF) as well as cellular component (CC). Besides, *P* < 0.05 served as the screening threshold. The first nine GO analysis items and KEGG pathways were screened from the results.

### 2.4. Survival Analysis

In this study, we adopted Cox proportional hazards model to identify prognostic DEGs (acquired in TCGA). *P* < 0.01 indicated significance.

### 2.5. Human Protein–Protein Interaction (PPI) Analysis, Hub Gene Identification, and Prognosis

This study retrieved PPI network based on the STRING database [17–19], which was reconstructed by applying Cytoscape (version 3.7.1) [17–19], and the threshold of the interaction was ≥0.15. Besides, module analysis on PPI network was conducted based on the plug-in Molecular Complex Detection (MCODE) [17–19]. The parameters were denoted as follows: degree cut − off > 5 and the number of adjacent nodes (*k* − core) > 5. The rest of the settings were default. The GO enrichment analysis was conducted using the obtained key genes as the enrichment background through Metascape (http://metascape.org/gp/index.html#/main/step1).

For the purpose of verifying the prognostic value of DEGs, this study applied the Kaplan–Meier Plotter (http://kmplot.com/analysis/) for deeply analyzing existing association of magnification of these genes with overall survival (OS) of those with gastric cancer. Apart from that, we classified cases as the following 2 groups in accordance with median gene expression (high or low level). Oncomine (https://www.oncomine.org/resource/main.html) is regarded as the largest tumor microarray database and comprehensive data analysis platform worldwide that is aimed at mining genetic information for cancer. To further analyze the difference in hub gene levels within GC, this study chose gene levels in GC reported by Wang et al. [20], Cho et al. [21], and D'Errico et al. [22] in the Oncomine database and visualised them using GraphPad Prism. We employed GEPIA (http://gepia.cancer-pku.cn/detail.php) for deeply confirming the differential hub gene levels. Additionally, *P* < 0.05 stood for significance.

### 2.6. Gene Set Enrichment Analysis (GSEA)

GNB4 expression showed that the samples from the complete cohort of TCGA were divided as 2 groups (high or low risk). The grouping was conducted with the use of GSEA of Sangerbox. Enrichment analysis was conducted using the KEGG gene set biological process database (c2.cp.kegg.v6). In addition, the *P* < 0.01 with the false discovery rate (FDR) is indicated as <0.01.

### 2.7. Immune Mechanism of GNB4

As a friendly portal website, TISIDB (http://cis.hku.hk/TISIDB/) combines a total of 988 immune antitumor genes from seven databases [23]. The association of immune characteristics with one gene is investigated based on 30 TCGA-derived cancers. We applied the TISIDB database in the present work for investigating the association of GNB4 level (or methylation) with tumor-infiltrating lymphocytes (TILs). *P* < 0.05 indicated significance.

As a comprehensive website, TIMER (https://cistrome.shinyapps.io/timer/) can comprehensively analyze immune infiltration of a total of 10,897 tissues of 32 cancers [24]. Moreover, the seq profiles for immune cells within tumor tissues can detect and quantify the infiltration in tumor tissue and determine the relation of tumor with immune cells. Moreover, we adopted this database for accurately quantifying the purity and immune infiltration level of tumors and assess the association of gene level with immune cell markers. Apart from that, the *P* < 0.05 and correlation coefficient |*R*| > 0.4 were regarded meaningful.

### 2.8. Quantitative Real-Time PCR (qRT-PCR)

GC together with adjacent healthy samples were exposed to Trizol reagent (TaKaRa Bio Inc. Shiga, Japan) to extract total RNA. cDNA was synthesised based on the PrimeScript™ RT Kit (TaKaRa, RR036A). In this work, real-time fluorescence quantitative PCR (qRT-time PCR) was performed based on the TBRreen™GReen™TremexExTaq™ Kit (TaKaRa, RR420A). The primers used were as follows: GNB4: sense strand 5′-GGTGATGACCTGTGCTTAT-3′ and antisense strand 5′-CAACTCTCGGCTTACTCTC-3′; GAPDH: sense strand 5′-GTCAACGGATTTGGTCTGTATT-3′ and antisense strand 5′-AGTCTTCTGGGTGGCAGTGAT-3′.

The Institutional Medical Ethics Review Committee of Taizhou First People's Hospital of Zhejiang Province supported the exploration in its facilities with the ethical application literature of 2022-KY001-01.

### 2.9. Determination of Tumor-Infiltrating Lymphocytes

The lymphocytes in the tumor stroma were evaluated, the tumor-infiltrating lymphocytes (TILs) at the boundary position were evaluated, and the tertiary lymphatic structure of extratumoral and intraepithelial TILs was not evaluated. The areas of necrosis or fibrosis were excluded, and only the area ratio of monocytes (including lymphocytes and plasma cells) to stroma was calculated. The whole visual field was scanned under a low-power microscope, and the representative areas were selected; 10 visual fields under 400x magnification were chosen for evaluation, and the average value was considered. The grouping was conducted according to the area ratio of TIL to stroma.

### 2.10. Statistical Analysis

IBM SPSS22.0 software (Chicago, IL) was adopted for exploring the association between clinicopathological phenotype and stromal/immune score of gastric cancer. We adopted the Mann–Whitney *U* test to compare stromal/immune scores of different clinicopathological groups. The Kaplan–Meier method was employed for the assessment of the overall survival rate. The log-rank test was employed for contrasting distinction of OS between high- and low-scoring groups. This work employed Spearman correlation for investigating the association of GNB4 mRNA expression with TILs. GNB4 mRNA expression data are denoted as mean ± standard deviation. One-way ANOVA was adopted for constant variables. *P* < 0.05 stood for significance.

## 3. Results

### 3.1. Correlation among Stromal Score/Immune Score, Clinical Characteristics, and Prognosis

Overall, a total of 338 cases were divided as 2 groups in accordance with Lauren's classification [25] of GC, respectively, intestinal GC, diffuse GC, and other cancer types. Patients with diffuse gastric cancer are poorly differentiated and have a worse prognosis. A comparison between the stromal score/immune score of the three groups of gastric cancer indicated that the stromal score/immune score of patients with diffuse gastric cancer was high (*P* < 0.001; Figures 1(b) and 1(c)). The difference in the stromal score/immune score of early GC (stage I) and advanced GC (stages II + III + IV) was analysed using the same method. Advanced gastric cancer exhibited a high stromal score/immune score (*P* < 0.001), as shown in Figures 1(d) and 1(e). The underlying association of the OS rate with stromal/immune score was investigated by dividing 349 patients with gastric cancer as a high- or low-score group, which can be found in Figures 1(f) and 1(g). Patients having a low stromal score had higher median OS rate when compared with that of patients with a high stromal score (1095 vs. 779 days, log-rank test, *P* = 0.017). Likewise, those who had a low immune score had higher OS rate than that of patients acquiring a high immune score (1294 vs. 588 days, log-rank test, *P* = 0.024). Obvious distinctions were detected. Moreover, the survival rate of patients undergoing high stromal/immune scores obviously decreased compared with those acquiring low stromal and immune scores (558 vs. 1294 days, log-rank test, *P* = 0.003) (Figure 1(h)).

### 3.2. DEG Identification in Gastric Cancer

**Figure 2:**
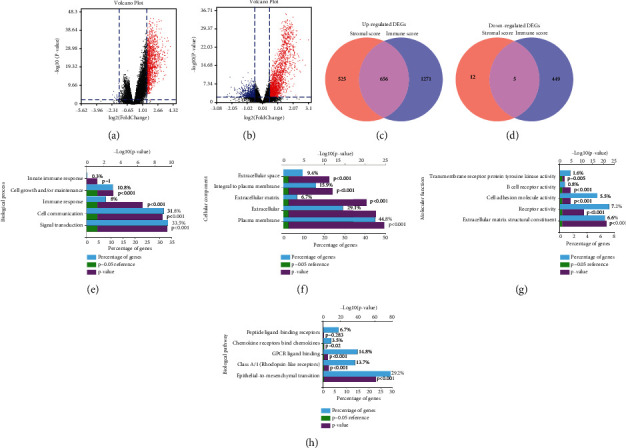
Expression profiles and biological functions of DEGs based on stromal and immune scores. (a) Volcano plot showing upregulated DEGs in red and downregulated DEGs in blue for the comparison based on high and low stromal score groups. (b) Volcano plot showing upregulated DEGs in red and downregulated DEGs in green for the comparison based on high and low immune score groups. (c, d) Venn diagrams showing 656 shared upregulated DEGs (c) and 5 shared downregulated DEGs (d) from stromal score and immune score groups. (e–h) Top ten GO terms and pathways enriched by DEGs.

After standardising the RNA-seq data of a total of 338 cases undergoing gastric cancer acquired from TCGA database, they were compared with the low-score group. According to the comparison of stromal score, a total of 1181 genes experienced upregulation with 17 genes being downregulated (Figure 2(a)). In accordance with the comparison of immune scores, a total of 1927 genes experienced upregulation with 454 genes being downregulated in the high-score group (Figure 2(b)). Based on Venn diagram (c) and (d) analyses, 656 shared the upregulated DEGs and 5 shared the downregulated DEGs within stromal/immune score groups and were chosen in this study for performing the follow-up analysis.

### 3.3. Functional and Pathway Enrichment Analyses

This work carried out GO and pathway enrichment analysis on the above-mentioned 656 upregulated genes and 5 downregulated genes (Figures 2(e)–2(h)). As for BP, DEGs were mainly related to innate immune response, cell growth and/or maintenance, immune response, cell communication, and signal transduction. In terms of CC, DEGs were mostly associated with extracellular space, integral to plasma membrane, extracellular matrix, extracellular, and plasma membrane. MF analysis was mainly associated with transmembrane receptor protein tyrosine kinase activity, B cell receptor activity, cell adhesion molecule activity, receptor activity, and extracellular stromal structural constituent. Based on pathway enrichment analysis, the genes were mostly involved in peptide ligand-biding receptors, chemokine receptor-binding chemokines, GPCR ligand binding, class A/1 (rhodopsin-like receptors), and epithelial-to-mesenchymal transition.

### 3.4. Identification of Prognostic DEGs in Gastric Cancer

**Figure 3:**
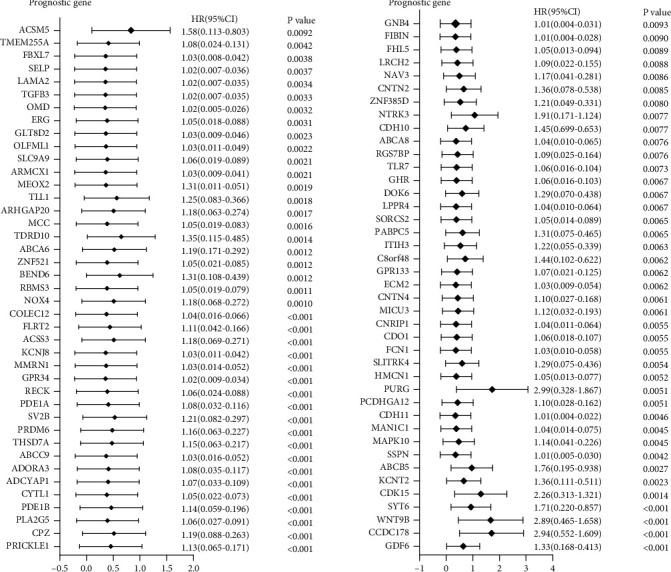
Forest plot of hazard ratios (HR) for 82 prognostic DEGs in gastric cancer. HR and 95% confidence intervals (CI) were obtained by the Cox proportional hazards model. HR and 95% confidence intervals (CI) were obtained by the Cox proportional hazards model.

For the purpose of determining the potential DEGs related to gastric cancer prognosis, this study built the Cox proportional model. Among those 656 upregulated and 5 downregulated indices, 82 upregulated indices predicted the dismal GC survival (Figure 3).

### 3.5. PPI Network Establishment as well as Hub Gene Analysis of Those Prognosis-Related DEGs and Hub Gene Prediction Value

**Figure 4:**
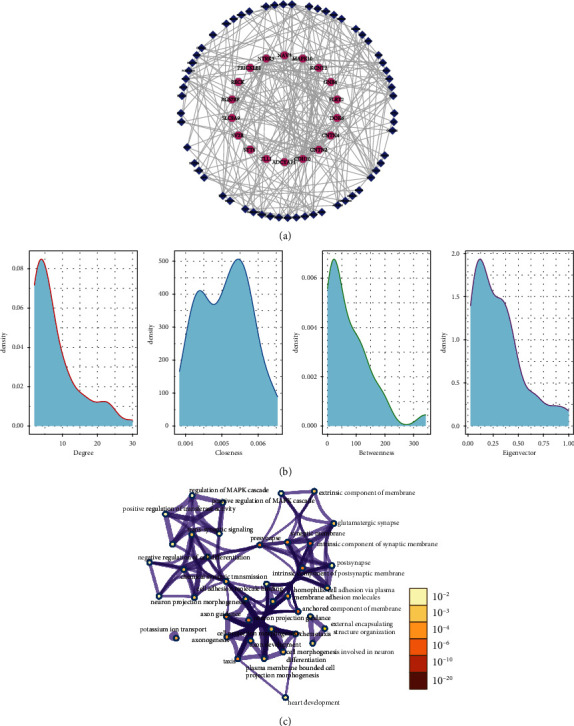
PPI network, GO analysis, and expression of hub gene. (a) PPI network that contained 77 nodes and 239 edges based on STRING tool and Cytoscape software was constructed. The hub genes were selected by MCODE in rose red with 18 nodes and 50 edges. (b) Topological properties and distribution of networks. (c) The GO analysis of hub genes was performed using Metascape. The color of the node represented the corrected *P* value of ontologies. *P* < 0.01 was considered statistically significant.

For the purpose of investigating the association between 82 identified prognostic DEGs, this study built the PPI network with 239 edges and 77 nodes with the application of the Cytoscape software and STRING tool. MCODE was adopted for performing module analysis with the selection of 18 hub genes. These genes constituted the PPI network that contained 50 edges and 18 nodes, as shown in Figure 4(a). We analyzed the degree distribution, closeness centrality, betweenness centrality, and eigenvector centrality distribution of the network (Figure 4(b)). It can be observed that the network presents the characteristics of dark rate distribution, which is in line with the characteristics of biological network. Completely comprehending the biological functions of the above-mentioned hub genes may be helpful in clarifying their potential mechanism in gastric cancer. GO biological process enrichment analysis was carried out by adopting Metascape for the 18 hub genes (Figure 4(c)). Based on the obtained results, 18 hub genes were mostly associated with the intrinsic component of synaptic membrane, presynapse, and axon guidance.

**Figure 5:**
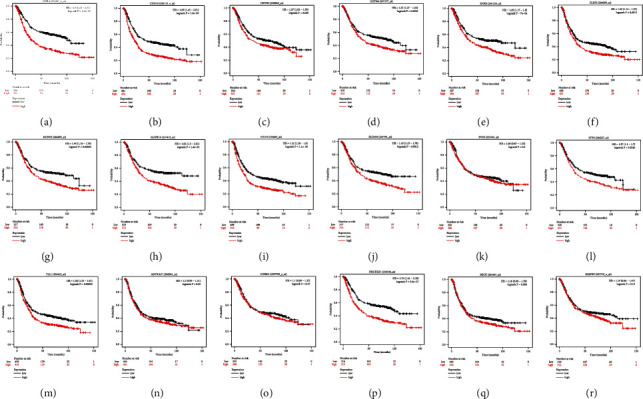
The prognostic value of mRNA level of hub genes in gastric cancer patients (*n* = 881) (Kaplan-Meier Plotter). Generally, higher mRNA expressions of were significantly associated with shorter OS of gastric cancers patients (a–p). However, NTRK3 and SV2B mRNA expressions showed no correlation with prognosis in gastric cancer patients (q, r).

In this work, we adopted the Kaplan–Meier Plotter tool for investigating the connection of hub gene mRNA expression with survival rate of totally 881 patients undergoing gastric cancer. According to Figure 5, most hub gene mRNA levels were in significant association with GC survival, except for NTRK3, SV2B, ADCYAP1, RECK, and RGS7BP.

**Figure 6:**
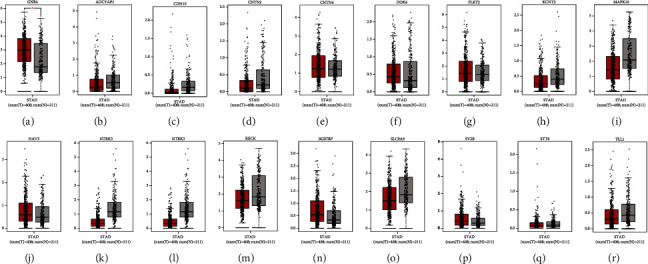
The expressions of the hub genes in GC patients (GEPIA). Box plots derived from gene expression data for GEPIA comparing the expression of hub genes in GC tissues and normal tissues (a–r); the *P* value was set at 0.01. Tumor tissue is shown in red, and normal tissue is shown in gray. ∗The results are statistically significant.

**Figure 7:**
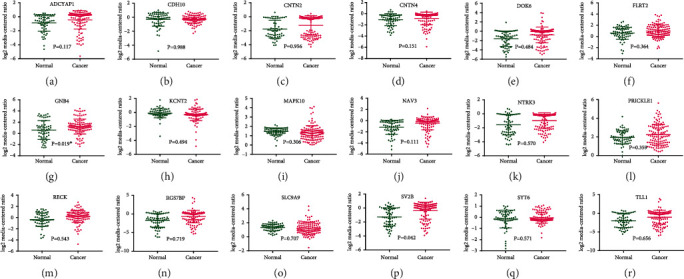
Expressions of different hub genes in GC cancers (Oncomine database). Gene expression data for Oncomine comparing the expression of hub genes in GC tissues and normal tissues (a–r). The scatter plot was drawn by GraphPad Prism 7. Tumor tissue is shown in orange, and normal tissue is shown in green. *P* < 0.05 was considered statistically significant.

This work explored the distinction in the expression of mRNA of 18 hub genes within GC samples (408 cases) as well as healthy samples (211 cases) with the GEPIA online tool. The results demonstrated that GNB4 was strongly denoted within GC tissues, with significant difference (Figure 6). To further understand central genes in gastric cancer, this study employed mRNA data in the Oncomine database for the purpose of investigating the mRNA expression level of hub genes (Figure 7). When compared with common tissues, 2 of the 18 hub genes were significantly upregulated in gastric cancer tissues (including GNB4 and SV2B). In conclusion, the hub gene GNB4 can be further studied as a molecular marker for clinically treating and preventing gastric cancer.

### 3.6. GSEA Based on TCGA Database

**Figure 8:**
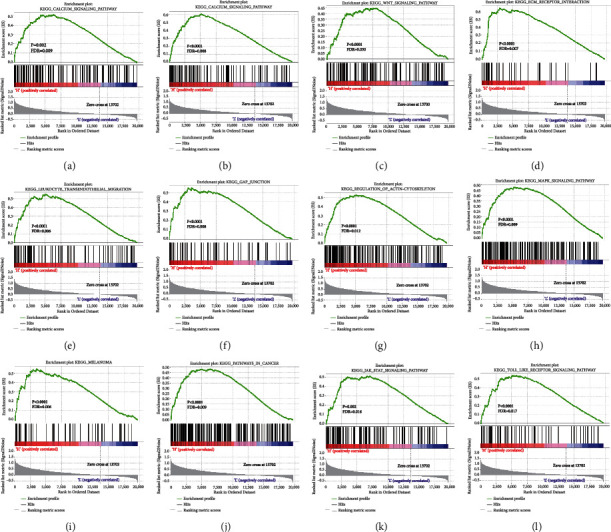
GSEA enrichment plots showed that eleven gene sets related to tumor signaling pathways (calcium signaling pathway (a), cell adhesion molecules (CAMs) (b), wnt signaling pathway (c), ecm receptor interaction (d), leukocyte transendothelial migration (e), gap junction (f), regulation of actin cytoskeleton (g), MAPK signaling pathway (h), melanoma (i), pathways in cancer (j), JAK-STAT signaling pathway (k), and toll-like receptor signaling pathway (l)) were enriched in the high GNB4 expression group.

With the aim to deeply analyze GNB4's effect on GC, this current work performed the KEGG pathway enrichment analysis based on GSEA. GNB4 enriches 72 gene sets, including 12 gene sets associated with cancer-related processes (Figure 8). Apart from that, GNB4 upregulation was possibly related to “cell adhesion molecules (CAMs)” (NES = 2.12, *P* = 0.002, FDR = 0.012), “cytokine–cytokine receptor interaction” (NES = 2.04, *P* < 0.000, FDR = 0.004), “chemokine signalling pathway” (NES = 2.00, *P* = 0.005, FDR = 0.006), “T cell receptor signalling pathway” (NES = 1.80, *P* = 0.004, FDR = 0.026), “B cell receptor signalling pathway” (NES = 1.81, *P* = 0.002, FDR = 0.025), “natural killer cell-mediated cytotoxicity” (NES = 1.83, *P* = 0.006, FDR = 0.022), “Fc epsilon ri signalling pathway” (NES = 1.85, *P* < 0.001, FDR = 0.019), “inositol phosphate metabolism” (NES = 1.73, *P* = 0.008, FDR = 0.032), “aldosterone regulated sodium reabsorption” (NES = 1.79, *P* = 0.002, FDR = 0.026), “insulin signalling pathway” (NES = 1.75, *P* < 0.001, FDR = 0.031), “calcium signalling pathway” (NES = 2.05, *P* = 0.002, FDR = 0.004), “complement and coagulation cascades” (NES = 1.71, *P* = 0.004, FDR = 0.035), “vascular smooth muscle contraction” (NES = 2.09, *P* = 0.002, FDR = 0.007), “focal adhesion” (NES = 1.98, *P* < 0.001, FDR = 0.007), and “leukocyte transendothelial migration” (NES = 2.06, *P* < 0.001, FDR = 0.004). Based on the obtained results, immunity and metabolism might also become the underlying mechanism for involving GNB4 in both the incidence and progress of gastric cancer.

### 3.7. Regulation of Immune Molecules by GNB4

**Figure 9:**
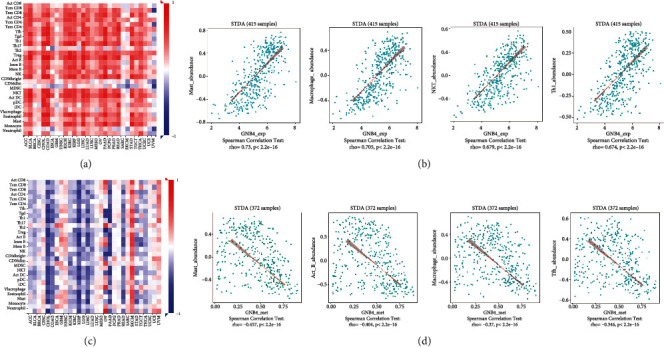
Spearman's correlations between GNB4 and lymphocytes (TISIDB). (a) Relations between the GNB4 expression and abundance of TILs across human cancers. (b) Top four greatest positive correlations between GNB4 expression and TILs. (c) Relations between the GNB4 methylation and abundance of TILs across human cancers. (d) Top four greatest negative correlations between GNB4 methylation and TILs.

As suggested by enrichment analysis, immune-cell-related pathway and cytokine–cytokine receptor interaction are involved notably; this study investigated whether immune cell GNB4 infiltration is engaged in gastric cancer (GC) pathogenesis. On the basis of the TISIDB database, the association between GNB4 expression, methylation, and lymphocytes was analyzed (Figure 9). Besides, the connection between GNB4 expression and the types of TILs in immune-related features is shown in Figure 9(a).  Figure 9(b) shows the largest correlation coefficients for mast cells (*r* = 0.73, *P* < 2.2*e*^−16^), natural killer T cells (NKT; *r* = 0.679, *P* < 2.2*e*^−16^), and macrophages (*r* = 0.705, *P* < 2.2*e*^−16^), as well as type 1 T-helper cells (Th1; *r* = 0.674, *P*1 < 2.2*e*^−16^). The correlation between methylation of GNB4 and lymphocytes can be found in Figure 9(c).  Figure 9(d) shows significant negative correlations for cells, including mast cells (*r* = −0.457, *P* < 2.2*e*^−16^), Act-B cells (*r* = −0.404, *P* < 2.2*e*^−16^), macrophages (*r* = −0.37, *P* = 2.23*e*^−13^), and T follicular helper cells (Tfh; *r* = −0.346, *P* = 9.44*e*^−12^). As a result, GNB4's mechanism within GC was possibly related to TIL modulation.

**Figure 10:**
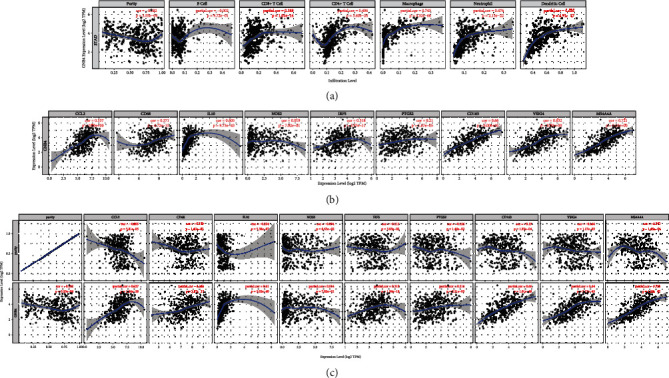
Correlations of GNB4 expression with immune infiltration level in STAD. (a) GNB4 expression is correlated with the level of immune infiltration in gastric cancer. (b) Correlation between GNB4 expression and the gene markers of monocytes (CCL2, CD68, and IL-10); M1 (NOS2, IRF5, and PTGS2); M2 (CD163, VSIG4, and MS4A4A) without adjustment. (c) Correlation between GNB4 expression and the gene markers of monocytes (CCL2, CD68, and IL-10); M1 (NOS2, IRF5, and PTGS2); M2 (CD163, VSIG4, and MS4A4A) adjusted by purity.

This study applied the TIMER database for studying connection of GNB4 expression with infiltrating immune cells within gastric cancer. According to the obtained results, GNB4 expression was most strongly related to macrophages (Cor = 0.743, *P* = 3.32*e*^−66^) with CD4^+^ T cells (Cor = 0.496, *P* = 3.60*e*^−24^), dendritic cells (Cor = 0.656, and *P* = 4.91*e*^−47^), as well as neutrophils (Cor = 0.476, *P* = 2.13*e*^−22^) (Figure 10(a)). As the purity of tumor in clinical samples affects the analysis of immune infiltration, we adjusted the purity of correlation analysis. GNB4 expression was notably related to the tumor-associated macrophage (TAM) markers CCL2 (Cor = 0.527, *P* = 1.64*e*^−28^), IL10 (Cor = 0.61, *P* = 5.02*e*^−40^), M2 macrophage marker CD163 (Cor = 0.66, *P* = 1.01*e*^−48^), VSIG4 (Cor = 0.64, *P* = 5.17*e*^−45^), and MS4A4A (Cor = 0.723, *P* = 1.26*e*^−62^). However, GNB4 expression exhibited a weak correlation with the M1 macrophage markers NOS2 (Cor = 0.014, *P* = 7.82*e*^−01^), IRF5 (Cor = 0.319, *P* = 1.94*e*^−10^), and PTGS2 (Cor = 0.216, *P* = 2.31*e*^−05^) (Figures 10(b) and 10(c)).

### 3.8. The Expression of GNB4 mRNA and Association of GNB4 mRNA Level with TILs

**Figure 11:**
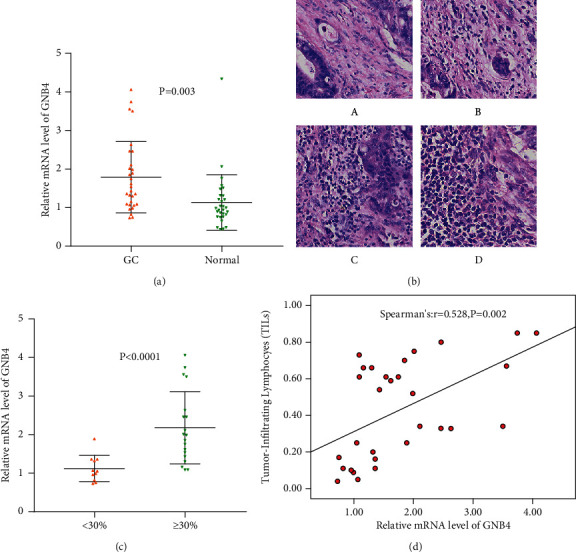
Evaluation of TILs and GNB4 mRNA levels in gastric cancer patients. (a) The relative mRNA expression levels of GNB4 in gastric cancer tissues and adjacent normal tissues were confirmed by qRT-PCR. (b) H/E sections (magnification ×100) from 4 cases of operable gastric cancer evaluated for TILs as follows: 2%, 20%, 40%, and 90%. (c) Scatter plot representing the expression of GNB4 mRNA levels related to TIL groups of gastric cancer patients. (d) Spearman correlation analysis of GNB4 mRNA levels with TILs.

GNB4 expression was verified on 30 GC and matched noncarcinoma samples by adopting qRT-PCR. GNB4 mRNA level was compared with the normal tissues adjacent to cancer cells in patients undergoing gastric cancer. The former was obviously higher in comparison with that in the latter (*P* = 0.003) (Figure 11(a)).

According to the percentage of TILs, the patients (*n* = 30) were divided into low TIL cohort (11 patients; 36.7%) < 30% (Figures 11(b) A and B) and high TIL cohort (19 patients; 63.3%) ≥ 30% TILs (Figures 11(b) C and D). The percentage of stromal TILs was significantly correlated with GNB4 mRNA expression. The patients with high TIL percentage (≥30%) expressed higher GNB4 mRNA levels than those with weak TIL infiltration (<30%) (*P* < 0.001; Figure 11(c)). Spearman's correlation demonstrated association of the tumor GNB4 mRNA level with TILs (*r* = 0.528, *P* = 0.002; Figure 11(d)).

## 4. Discussion

Cancer has been considered the main cause of death globally. Common therapies of cancer consist of surgery, chemotherapy, and radiotherapy [26, 27]. Cancer immunotherapy has achieved great success with studies on the immune molecular mechanisms in cancer occurrence and development. Blocking immune checkpoints, TILs, and immunotherapy of T cell receptor chimeric T cells and chimeric antigen receptor T cells have obtained some achievements in the therapy for different tumors, especially lung cancer, cutaneous malignant melanoma, and B cell lymphoma [28–30]. However, only 10%–20% of patients benefit from these treatments [31, 32]. Therefore, new and more immune-related therapeutic targets of gastric cancer should be urgently clarified and identified.

In the research, the ESTIMATE algorithm was adopted to analyze gene levels in TCGA-derived GC cases. The high and low immune scores as well as stromal scores of all the involved patients were acquired. Further, we screened 661 DEGs related to the tumor microenvironment. These genes were found to be primarily involved in signal transduction (BP), receptor activity (MF), and plasma membrane (CC), as well as epithelial mesenchymal transformation (pathway). Thus, GNB4, ACDH10, ADCYAP1, PRICKLE1, CNTN2, SV2B, MAPK10, DOK6, NTRK3, CNTN4, FLRT2, KCNT2, RGS7BP, TLL1, SYT6, NAV3, SLC9A9, and RECK were considered prognostic hub genes. Kaplan–Meier Plotter online database analysis suggested that all prognostic hub genes except NTRK3 and SV2B were in a significant relationship with patient's overall survival. Oncomine and GEPIA database analysis revealed that gastric cancer tissues had higher GNB4 expression compared with normal gastric mucosa (*P* < 0.01 and *P* = 0.019, respectively). We further studied the underlying mechanism for GNB4 in gastric cancer by GSEA, observing the potential involvement of high GNB4 expression in tumor-related signalling pathways and immune and metabolic processes. TISIDB database research uncovered positive association of GNB4 level with the highest extent with TILs (macrophages and mast, NKT, and Th1 cells), and the methylation of GNB4 was negatively correlated to the highest extent with TILs (macrophages and mast, Act-B, and Tfh cells). The TIMER database showed the significant positive correlation between GNB4 expression and TILs (macrophages, CD4+ T cells, dendritic cells, and neutrophils, as well as CD8+ T cells). Many studies have reported the positive association of high TILs level with survival of multiple tumors, like breast cancer (BC) [33] or non-small-cell lung cancer (NSCLC) [34], which improves the curative effect of neoadjuvant chemotherapy in breast cancer [35]. In addition, TILs are known as predictive prognostic markers for gastric cancer [36, 37]. However, some studies have shown that TILs exhibit no correlation with gastric cancer prognosis [38].

GNB4 is one of the three subunits of heterotrimeric G-protein *β* subunit. It is located at 3q26.33 and is composed of 12 exons. It mainly transduces the upstream signal of G-protein-coupled receptor to the downstream pathway to regulate the cancer cell biological behaviour [39]. The haplotype block in intron 1 of GNB4 is significantly related to bladder urothelial carcinoma development and prognosis [39]; the high GNB4 expression is in a significant correlation with the survival rate in patients having breast cancer or colorectal cancer [40, 41]. Obviously, in gastric cancer, GNB4 possibly acts as a biomarker for abnormal methylation, playing a vital function in the growth and metastasis of *Helicobacter pylori*-induced gastric cancer [42]. Furthermore, GNB4 expression was in a significant correlation with gastric cancer patients' pathological stage and tumor invasion depth, as well as survival rate; in vitro tests verify the positive effect of GNB4 overexpression during epithelial−mesenchymal transformation (EMT) [43]. The qRT-PCR results of the research indicate the remarkably elevated GNB4 expression of gastric cancer tissues (*P* = 0.003), in agreement with former studies. Additionally, we observed the significant positive correlation between GNB4 mRNA expression and TILs level. To sum up, GNB4 exerts major effects on the malignant biological behaviour of tumors, especially in gastric cancer. GNB4 can regulate the antitumor immune response while promoting GC growth and progression. Thus, GNB4 is an underlying factor to predict the prognosis and treat GC.

**Figure 12:**
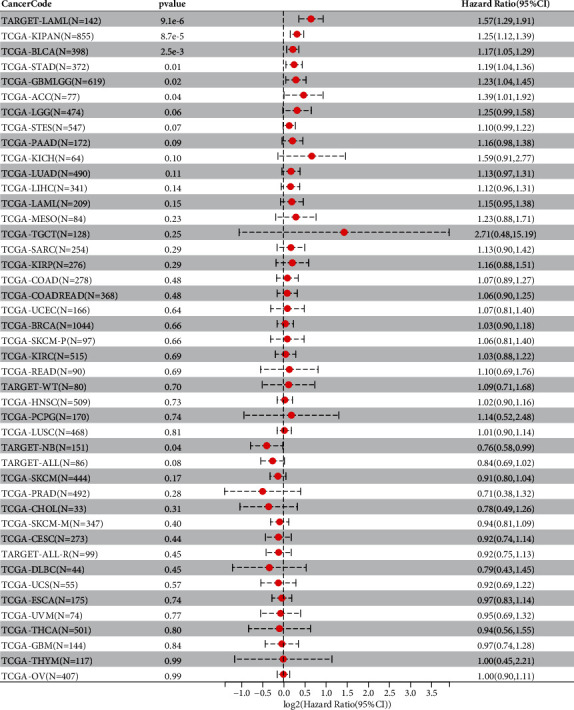
Correlation between G expression in pancancer and prognosis.

Though GNB4's clinical importance and function in gastric cancer have been reported, the research verified the potential use of GNB4 as a prognostic biomarker and provided some ideas for studying the potential mechanism of GNB4 involved in tumor immune cell infiltration. In addition, it can be observed that GNB4 is not only related to gastric cancer but also significantly related to the adverse prognosis of leukemia, bladder cancer, glioma, etc. (Figure 12). GSEA indicated that GNB4 is involved in various tumor-related pathways, including calcium signalling pathway, cell adhesion molecules cams, regulation of actin cytoskeleton, gap junction, leukocyte transendothelial migration, MAPK pathway, melanoma, pathways in cancer, JAK-STAT pathway, toll-like receptor pathway, ecm receptor interaction, and wnt pathway. In addition, GNB4 is involved in various immune and metabolic pathways, including cytokine–cytokine receptor interaction, CAMs, chemokine signalling pathway, NK cell-mediated cytotoxicity, T cell receptor pathway, B cell receptor pathway, Fc epsilon ri pathway, inositol phosphate metabolism, aldosterone-regulated sodium reabsorption, insulin pathway, complement and coagulation cascades, calcium pathway, vascular smooth muscle contraction, focal adhesion, and leukocyte transendothelial migration. TISIDB and TIMER databases suggested that GNB4 level was significantly related to macrophages (in lymphocytes). Tumor-associated macrophages (TAMs) is a major element in gastric cancer TME cells. They have been reported to greatly promote tumor growth and metastasis [44]. TAM is a major participant in tumor-related inflammation, which exerts significant effects on tumor proliferation and migration, immunosuppression, and neovascularisation. In all these processes, M1 macrophages play a crucial role in recognising and attacking tumor cells, whereas M2 macrophages exert significant effects on immunosuppression and tumor progression [45]. Besides, we observed that GNB4 can aggregate macrophage infiltration and is significantly positively correlated with TAM markers (CCL2, CD68, and IL10), particularly M2 macrophage markers (CD163, VSIG4, and MS4A4A). Thus, we speculate that GNB4 is related to M2 TAM infiltration. Therefore, GNB4 suppression possibly reduces TAM infiltration, particularly M2 TAM. It can further improve the response of T cells, providing a new idea for immunotherapy against gastric cancer.

The research is limited in some aspects. The data are based on database analysis. Although high GNB4 expression of gastric cancer tissues was confirmed through qRT-PCR, in vivo and in vitro research and more prospective clinical trials should be performed to verify GNB4's role in gastric cancer. Furthermore, our results illustrate GNB4's key effect on GC immune environment. In-depth explorations should be carried out to further verify the potential mechanism of GNB4 interaction with specific immune markers and immune cells.

## 5. Conclusions

In conclusion, GNB4 is a gene which shows association with the immune microenvironment of gastric cancer. This predicts poor GC survival. According to bioinformatic analysis, GNB4 is involved in tumor-related signalling pathways and immune and metabolic processes. Thus, the study offers a novel target to investigate the underlying mechanism for gastric cancer.

## Figures and Tables

**Table 1:** Clinical characteristics of TCGA.

TCGA (*n* = 338)	
Survival state	
Dead	138 (40.8%)
Alive	200 (59.2%)
Gender	
Female	120 (35.5%)
Male	218 (64.5%)
TNM stage	
I	46 (13.6%)
II	106 (31.4%)
III	137 (40.5%)
IV	35 (10.4%)
Unknown	14 (4.1%)
Lauren's classification	
Intestinal GC	271 (80.2%)
Diffuse GC	67 (19.8%)

## Data Availability

Data generated through this work could be requested from the corresponding author via reasonable request.

## Additional Points

*Reporting Checklist*. The authors have completed the REMARK reporting checklist. *Open Access Statement*. This is an Open Access article distributed in accordance with the Creative Commons Attribution-NonCommercial-NoDerivatives 4.0 International License (CC BY-NC-ND 4.0), which permits the noncommercial replication and distribution of the article with the strict proviso that no changes or edits are made and the original work is properly cited (including links to both the formal publication through the relevant DOI and the license) (see: https://creativecommons.org/licenses/by-nc-nd/4.0/).
